# Genome-Wide Detection of Selective Signature in Chinese Holstein

**DOI:** 10.1371/journal.pone.0060440

**Published:** 2013-03-28

**Authors:** Dunfei Pan, Shengli Zhang, Jicai Jiang, Li Jiang, Qin Zhang, JianFeng Liu

**Affiliations:** Key Laboratory of Animal Genetics, Breeding and Reproduction, National Engineering Laboratory for Animal Breeding, College of Animal Science and Technology, Ministry of Agriculture, China Agricultural University, Beijing, China; University of Queensland, Australia

## Abstract

Selective signatures in whole genome can help us understand the mechanisms of selection and target causal variants for breeding program. In present study, we performed Extended Haplotype Homozygosity (EHH) tests to identify significant core regions harboring such signals in Chinese Holstein, and then verified the biological significance of these identified regions based on commonly-used bioinformatics analyses. Results showed a total of 125 significant regions in entire genome containing some of important functional genes such as *LEP, ABCG2, CSN1S1, CSN3* and *TNF* based on the Gene Ontology database. Some of these annotated genes involved in the core regions overlapped with those identified in our previous GWAS as well as those involved in a recently constructed candidate gene database for cattle, further indicating these genes under positive selection maybe underlie milk production traits and other important traits in Chinese Holstein. Furthermore, in the enrichment analyses for the second level GO terms and pathways, we observed some significant terms over represented in these identified regions as compared to the entire bovine genome. This indicates that some functional genes associated with milk production traits, as reflected by GO terms, could be clustered in core regions, which provided promising evidence for the exploitability of the core regions identified by EHH tests. Findings in our study could help detect functional candidate genes under positive selection for further genetic and breeding research in Chinese Holstein.

## Introduction

When a novel beneficial mutation have been under the force of selection over a long period of time, it will show some obvious features such as unusually long-range linkage disequilibrium (LD) and a high population frequency, which therefore represents a detectable " signature of selection" [1], [2]. Identifying signatures of recent positive selection could help us target causal variants for breeding and be potential to provide straightforward insights into the mechanisms of evolution. Furthermore, it can possibly highlight the genetic basis of phenotypic diversity for complex traits [3], [4].

Many methods have been developed to detect such selection signals hitherto, such as the integrated Haplotype Homozygosity Score (iHS) and the extended haplotype homozygosity (EHH) test [5], [6] based on haplotype lengths; F_ST_ test [7] for population differentiation detection. These methods have been successfully implemented in many studies to exploit strong signatures of mutations under positive selection in humans and in domestic animals, *e.g.*, genes resistance to malaria [6], [8], in response to milk yield in dairy cattle [5], [9] and related to pigmentation [10].

In this study, we applied the EHH test to identify selective signatures in Chinese Holstein. The method of the EHH test, firstly introduced by Sabeti *et al.* (2002), is based on the relationship of an allele’s frequency and the extent of LD surrounding it. When under positive selection, it can make the allele frequency to increase at a very fast speed, such that the recombination does not totally break down the haplotype where the selected mutation occurs [6]. According to this, EHH is defined as the probability that two randomly chosen chromosomes carrying the core haplotype of interest are identical by descent for the entire interval from the core region to a certain point [6]. It has been reported that recombination rates are heterogeneous among chromosomal regions, and it may be a potential factor of false positives in the EHH detection [9]. REHH (relative EHH), proposed by Sabeti (2002), is supposed to overcome this limitation. The idea of REHH is to compare the EHH of the tested core haplotype with that of other core haplotypes for adjusting for local variation in recombination rates. With rectificatory recombination rates, we can readily use the REHH value to screen EHH at matched genetic distances, and haplotypes with excessive REHH values and high frequencies could indicate signal of positive selection [5], [6]. The superiorities of the EHH test are that it can incorporate information on both the allele frequency and the association among SNPs, and it is designed to work with SNP markers rather than sequencing data [9].

Since different populations are undergoing different climates, geographic environment and selective forces [11], the frequencies of alleles are largely different among populations as well as the selective signals [10]. Aiming at exploring specific selective signatures in Chinese Holstein, we performed a genome-wide detection for selective signatures employing EHH tests in present study. Our findings herein as well as incorporating those findings in our previous genome-wide association studies (GWAS) for milk production traits could possibly verify some specifically biologic functions reflected by these identified core regions, and provide foundations for further pinpointing causal variants for economically important traits in dairy cattle.

## Materials and Methods

### Animal Resource and Genotyping

A total of 2019 daughters and 87 sires from 15 Holstein cattle farms in Beijing were collected as experimental population. Samples were collected as part of the regular quarantine inspection of the farms, and used in this study with the permission of the animals' owners. The population structure has been detailed in our previous study [12]. DNA was extracted from blood (for daughters) and semen (for sires), and all individuals were genotyped using the Illumina BovineSNP50 BeadChip containing 54001 SNPs, with a mean distance of 48.75 kb between markers.

Before analyses, we used Beagle [13] to impute the missing genotypes and to construct haplotypes. Besides, quality control for SNP markers was performed to remove those with minor allele frequency (MAF) less than 0.03.

### Detection of Selection Signatures

For the EHH test, we firstly identified core regions in the genome using the software Sweep v.1.1 [6], and then calculated EHH values for haplotypes in each core region. The core region is characterized by SNPs with strong LD and consists of some core haplotypes [9]. To compare REHH values across many core features, genetic distance of 0.5 cM was chosen as the matched distance to determine the REHH value for each core region. The distance of 0.5 cM was an appropriate setting for the longer extent LD in cattle, as compared with that in humans [9], [14], [15].

### Bioinformatics Analyses

After carrying out EHH tests, we further performed multi-level bioinformatics analyses to explore potential biology significance of genes harbored in the identified core regions. The following two aspects were involved in the analyses:

#### Genome annotation

In the analysis, each significant core region was extended 1 Mb towards both sides as the target region for annotation. In addition, genes involved in the core regions were compared with those in our previous GWAS [12] as well as those involved in a database of candidate genes for milk production performance and mastitis [16].

#### Enrichment analyses

Two different types of enrichment analyses relevant to the significant core regions were performed herein, *i.e.*, gene ontology (GO) term enrichment analyses and pathway enrichment analyses. GeneMerge1.2 software tool [17] was employed to perform enriched GO term analyses and enriched pathway analyses.

## Results

### A Descriptive Statistics for Markers and Core Haplotype

After the quality control for SNP genotypes and excluding the SNPs on chromosome X, 40130 SNPs were finally retained in the analyses. Table 1 gives an overall description for the distribution of SNPs across whole genome. The numbers of SNPs for every chromosome ranged from 748 to 2610, with an average interval of 63.4 kb between adjacent markers.

**Table 1:** Summary of genome-wide marker and core region (CR) distribution in Chinese Holstein.

Chr	Chr length (Mbp)	SNP (n)	Mean distance (kb)	CR (n)	Total CR Length(kb)	Max CR Length(kb)	Mean CR Length(kb)	CR SNPs(n)^a^	Max CR SNPs(n)
1	161.06	2610	61.7	292	40305.4	628.64	138.0±90.2	1099	11
2	140.63	2109	66.7	228	32967.5	696.32	144.6±102.5	839	9
3	127.91	2025	63.2	222	32430.1	1073.97	146.1±117.3	820	9
4	124.13	1935	64.1	196	26006.6	509.61	132.7±90.2	709	9
5	125.80	1681	74.8	152	23513.1	561.02	154.7±100.6	554	12
6	122.54	1982	61.8	206	28214.8	558.29	137.0±99.9	774	10
7	112.06	1759	63.7	176	24310.3	1131.54	138.1±117.7	631	8
8	116.94	1851	63.2	190	26709.9	600.82	140.6±92.1	716	9
9	108.07	1570	68.8	155	20279.0	588.08	130.8±85.6	563	9
10	106.20	1683	63.1	173	20536.7	347.15	118.7±63.2	624	8
11	110.17	1780	61.9	194	23677.6	780.69	122.0±91.4	684	10
12	85.28	1290	66.1	120	16387.3	618.68	136.6±104.5	421	7
13	84.34	1373	61.4	142	18376.8	517.75	129.4±87.1	517	11
14	81.32	1360	59.8	152	21804.0	548.68	143.4±98.6	549	10
15	84.60	1324	63.9	136	19048.2	780.38	140.1±100.9	484	7
16	77.82	1231	63.2	134	17809.6	730.69	132.9±85.0	502	11
17	76.45	1250	61.2	127	15106.7	368.96	119.1±66.1	442	8
18	66.12	1072	61.7	84	10677.9	690.26	127.1±89.3	289	6
19	65.21	1106	59.0	91	11736.3	622.59	129.0±98.6	347	6
20	75.71	1250	60.6	115	14453.6	313.29	125.7±65.6	413	8
21	69.17	1072	64.5	100	12443.5	490.57	124.4±75.0	347	6
22	61.83	980	63.1	108	12539.8	487.54	116.1±66.6	364	6
23	53.33	870	61.3	79	9709.7	502.67	122.9±87.3	267	8
24	64.95	993	65.4	89	10595.9	518.43	119.1±86.6	302	7
25	44.02	790	55.7	76	7983.1	359.94	105.0±61.4	254	5
26	51.73	834	62.0	83	9855.6	463.48	118.7±67.7	288	6
27	48.73	774	63.0	64	7273.0	385.59	113.6±66.7	210	6
28	46.00	748	61.5	50	6432.7	324.42	127.8±61.7	167	5
29	51.98	828	62.8	62	7923.9	321.19	127.8±61.7	215	6
Total	2544.1	40130	63.4	3996	529108.6	16521.24	129.7±85.6	14364	12

a : the number of SNPs involved in core regions of each chromosome.

### Genome-wide EHH Tests

A total of 3,996 core regions were identified and corresponding 31,182 EHH tests have been performed. These identified core regions contained 14,363 SNPs, with a range of 3–12 SNPs per core. The total length of these cores was 529,108.6 kb, with a mean length of 129.7±85.6 kb.

**Figure 1:**
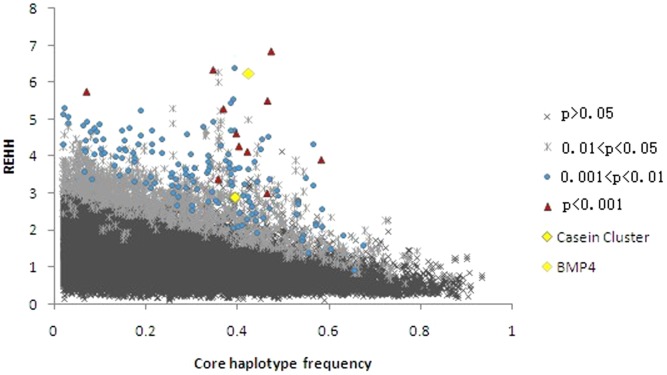
Distribution of the REHH vs. the core haplotype frequency. Different P values are marked by different color symbols presented in the right of this figure.

**Figure 2:**
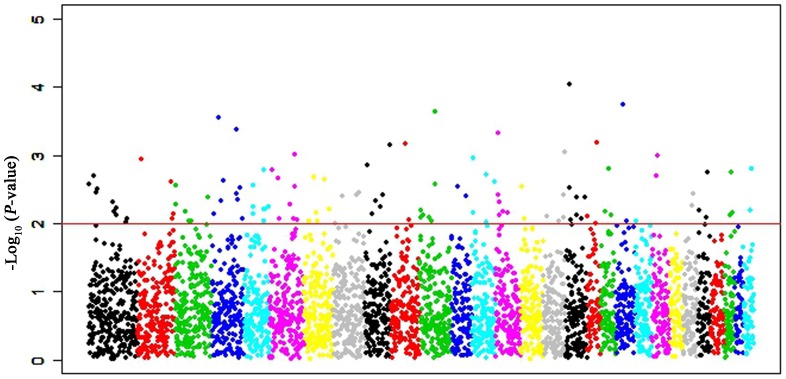
The distribution of the P values of haplotypes with frequency≥0.20 on the whole genome.

An intuitive scatter plot is presented in Fig. 1, showing the distribution of REHH values vs. haplotype frequencies. Different colors represented different ranges of *p* values. Moreover, a plot diagram is given in Fig. 2 to visualize the distribution of the REHH values across whole genome. Based on the concept of selective signature aforementioned, one character of such signals is the high frequency of a beneficial selected gene involved in a core haplotype. Hence we further discarded those core haplotypes with frequency <20%. Accordingly, 16,035 EHH tests remained for all core haplotypes after filtering (See Table 2), with a maximum of 1,177 tests on chromosome 1. Among these EHH tests, 541 and 125 out of them achieved significant level corresponding to the significance levels of 0.05 and 0.01, respectively.

**Table 2:** Summary of whole genome extended haplotype homozygosity tests.

Chr	Tests on CH (n)^a^	P-value<0.05 (n)	P-value<0.01(n)
1	1177	29	10
2	895	30	5
3	898	36	7
4	807	27	9
5	594	19	8
6	822	31	9
7	681	17	7
8	784	32	5
9	610	19	6
10	640	21	2
11	766	27	8
12	456	22	2
13	579	20	5
14	614	15	6
15	569	25	2
16	540	12	5
17	511	14	7
18	345	15	3
19	369	14	3
20	466	14	2
21	401	16	1
22	436	16	2
23	326	15	0
24	360	15	2
25	333	11	3
26	325	8	0
27	264	6	3
28	214	7	0
29	253	8	3
Total	16035	541	125

a : Core haplotypes involved in each core region determined by Sweep across genome.

### Genome Annotation

We annotated genes involved in all significant identified core regions based on the Gene Ontology database (http://www.geneontology.org/), and further found out those potential candidate genes relevant to milk production traits. Accordingly, a total of 9,829 genes had been identified, 6573 of them (66.87%) fall into the regions of QTL affecting milk production included in the cattle QTL database (http://www.animalgenome.org/cgi-bin/QTLdb/index).

In our previous GWAS [12], 75 genes were identified associated with one or multiple milk production traits in dairy cattle such as milk fat yield (FY), milk yield (MY), milk protein yield (PY), milk fat percentage (FP) and milk protein percentage (PP). Among these 75 genes, 45 out of them were contained in the core regions detected in present study, further indicating these genes under positive selection maybe underlie milk production traits in Chinese Holstein. Table 3 gives symbols of these genes as well as the closest core positions and corresponding *p* values of core haplotypes. Totally, 45 genes were involved in 19 core regions which contained 25 significant core haplotypes.

**Table 3:** Genes within the core regions overlapping with those by genome wide association studies by Jiang et al (2010).

Gene Symbol	Chr.	Closest core position (bp)	Hap Freq(%)	P-value
LOC614166	1	148796434–148911817	0.286	0.0547
		149189841–149242164	0.209	0.0365
DIP2A	1	148796434–148911817	0.286	0.0547
		149189841–149242164	0.209	0.0365
KBTBD10	2	27607855–27651437	0.382	0.0369/0.014
SLC30A7	3	44239453–44433532	0.373	0.0211
		45775675–45934869	0.418	0.00681
LOC511240	5	76788487–76882812	0.325	0.0154
		77546764–77805841	0.351	0.0587
HERC3	6	37135013–37231101	0.572/0.0479	0.00220/0.0378
PKD2	6	37135013–37231101	0.572/0.0479	0.00220/0.0378
		38479643–38558526	0.216	0.0562
NFIB	8	31713520–31832256	0.235	0.00359/0.00573
LOC788012	9	5992605–6049113	0.348/0.267	0.0334/0.0596
C14H8orf33	14	260341–443937	0.248	0.0568
FOXH1	14	260341–443937	0.248	0.0568
CYHR1	14	260341–443937	0.248	0.0568
VPS28	14	260341–443937	0.248	0.0568
DGAT1	14	260341–443937	0.248	0.0568
MAF1	14	260341–443937	0.248	0.0568
LOC786966	14	260341–443937	0.248	0.0568
GRINA	14	260341–443937	0.248	0.0568
		1889210–1967406	0.33	0.0454
GML	14	260341–443937	0.248	0.0568
		1889210–1967406	0.330	0.0454
		2163275–2239116	0.268	0.0554
GPIHBP1	14	1889210–1967406	0.330	0.0454
		2163275–2239116	0.268	0.0554
COL22A1	14	2805785–2849483	0.579	0.0556
		3018726–3099635	0.260	0.0401/0.0246
NKAIN3	14	28014144–28185224	0.322	0.0282
OPLAH	14	260341–443937	0.248	0.0568
MAPK15	14	260341–443937	0.248	0.0568
ZNF623	14	260341–443937	0.248	0.0568
EEF1D	14	260341–443937	0.248	0.0568
		1889210–1967406	0.330	0.0454
ZC3H3	14	260341–443937	0.248	0.0568
		1889210–1967406	0.330	0.0454
LYPD2	14	260341–443937	0.248	0.0568
		1889210–1967406	0.330	0.0454
RHPN1	14	1889210–1967406	0.330	0.0454
		2163275–2239116	0.268	0.0554
GPR20	14	1889210–1967406	0.330	0.0454
		2163275–2239116	0.268	0.0554
		2805785–2849483	0.579	0.0556
PTK2	14	1889210–1967406	0.330	0.0454
		2163275–2239116	0.268	0.0554
		2805785–2849483	0.579	0.0556
		3018726–3099635	0.260	0.0401/0.0246
EIF2C2	14	1889210–1967406	0.330	0.0454
		2163275–2239116	0.268	0.0554
		2805785–2849483	0.579	0.0556
		3018726–3099635	0.260	0.0401/0.0246
KCNK9	14	2163275–2239116	0.268	0.0554
		2805785–2849483	0.579	0.0556
		3018726–3099635	0.260	0.0401/0.0246
LOC618755	14	3018726–3099635	0.260	0.0401/0.0246
ZNF7	14	260341–443937	0.248	0.0568
EPPK1	14	260341–443937	0.248	0.0568
CYP11B2	14	260341–443937	0.248	0.0568
		1889210–1967406	0.330	0.0454
		2163275–2239116	0.268	0.0554
GLI4	14	1889210–1967406	0.330	0.0454
		2163275–2239116	0.268	0.0554
TRAPPC9	14	1889210–1967406	0.330	0.0454
		2163275–2239116	0.268	0.0554
		2805785–2849483	0.579	0.0556
		3018726–3099635	0.260	0.0401/0.0246
LOC782462	20	36691324–36837401	0.537/0.215	0.0187/0.0467
LOC782833	20	36691324–36837401	0.537/0.215	0.0187/0.0467
C9	20	36691324–36837401	0.537/0.215	0.0187/0.0467
FYB	20	36691324–36837401	0.537/0.215	0.0187/0.0467
RICTOR	20	36691324–36837401	0.537/0.215	0.0187/0.0467
LOC100138964	20	36691324–36837401	0.537/0.215	0.0187/0.0467
RAI14	20	40690535–40854433	0.341	0.0443

This table describes genes that associated with milk production traits when compared with genome wide association studies, Hap Freq describes the frequency of core haplotype involved in each core region determined by Sweep across genome.

**Figure 3:**
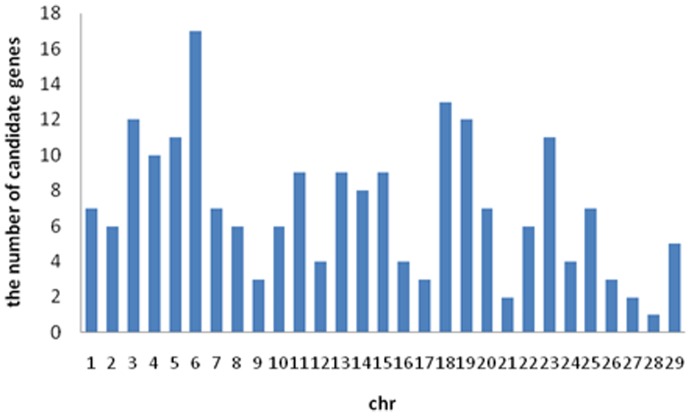
Distributions of candidate genes in whole genome.

In addition, 203 genes within the core regions overlapped with those included in a database of candidate genes in cattle underlying milk production performance and mastitis [16]. A histogram showing the overall distribution of the candidate genes across the genome is given in Fig. 3. It can be seen that the largest number of overlapped candidate genes were located on chromosome 6. Among these 203 functional genes, 13 genes (*LEP, ABCG2, CSN1S1, CSN3, IL8, TLR4, IL1B, LBP, DGAT1, TLR2, C5AR1, LTF, TNF*), which were suggested as being under selection, have been frequently reported in different studies [16].

### GO Term Enrichment Analyses

By searching for over-represented terms in core regions compared to those across whole genome, we can detect those functional genes clustered in the identified core regions contributing to milk production traits. We analyzed the significant enriched terms for genes involved in the core region. As a result, 36 second level GO terms (64.3%) were found to be significantly over-represented (Table 4). For instance, the term ‘metabolic process‘(GO: 0008152) showed significantly enriched, which indicated genes associated with milk production traits involved in ‘metabolic process‘ were clustered in the core regions. Other terms such as ‘immune system process‘(GO: 0002376) and ‘reproductive process‘(GO: 0022414) were also showed clear biological association with mastitis and milk production traits.

**Table 4:** Enriched GO terms when comparing candidate genes to the whole genome using GeneMerge1.2.

GO term	Description	Pop-frec	CR-frec	ratio	P value
GO:0000003	reproduction	210/27430	101/9829	1.34	9.08E-3
GO:0044421	extracellular region part	361/27430	171/9829	1.32	2.12E-4
GO:0016265	death	403/27430	182/9829	1.26	3.49E-3
GO:0022414	reproductive process	208/27430	101/9829	1.36	5.76E-3
GO:0032991	macromolecular complex	1327/27430	596/9829	1.25	1.03E-10
GO:0005623	cell	5024/27430	2264/9829	1.26	8.171E-49
GO:0048519	negative regulation of biological process	669/27430	306/9829	1.28	3.34E-06
GO:0050896	response to stimulus	1500/27430	693/9829	1.30	7.77E-16
GO:0044422	organelle part	1953/27430	885/9829	1.26	1.32E-17
GO:0051234	establishment of localization	1094/27430	494/9829	1.26	3.30E-09
GO:0031974	membrane-enclosed lumen	728/27430	340/9829	1.30	3.68E-08
GO:0022610	biological adhesion	198/27430	94/9829	1.32	0.0261
GO:0008152	metabolic process	2813/27430	1291/9829	1.28	1.81E-29
GO:0044464	cell part	5024/27430	2264/9829	1.26	8.17E-49
GO:0003824	catalytic activity	1991/27430	919/9829	1.29	2.99E-21
GO:0005488	binding	3393/27430	1534/9829	1.26	1.07E-31
GO:0009987	cellular process	3860/27430	1739/9829	1.26	1.49E-35
GO:0005215	transporter activity	400/27430	180/9829	1.26	4.84E-3
GO:0032502	developmental process	896/27430	409/9829	1.27	2.85E-08
GO:0002376	immune system process	390/27430	175/9829	1.25	7.19E-3
GO:0008283	cell proliferation	292/27430	142/9829	1.36	2.41E-4
GO:0005576	extracellular region	697/27430	320/9829	1.28	1.06E-06
GO:0023052	signaling	904/27430	424/9829	1.31	1.34E-10
GO:0048518	positive regulation of biological process	785/27430	359/9829	1.28	2.51E-07
GO:0032501	multicellular organismal process	1124/27430	494/9829	1.23	3.43E-07
GO:0051704	multi-organism process	189/27430	92/9829	1.36	0.0102
GO:0043226	organelle	3372/27430	1509/9829	1.25	1.65E-28
GO:0060089	molecular transducer activity	309/27430	151/9829	1.36	8.61E-05
GO:0050789	regulation of biological process	1900/27430	871/9829	1.28	5.19E-19
GO:0004872	receptor activity	347/27430	156/9829	1.25	0.0144
GO:0030234	enzyme regulator activity	261/27430	122/9829	1.30	9.57E-3
GO:0040011	locomotion	245/27430	115/9829	1.31	0.012
GO:0051179	localization	1269/27430	575/9829	1.26	4.01E-11
GO:0040007	growth	165/27430	81/9829	1.37	0.0170
GO:0071840	cellular component organization or biogenesis	1015/27430	474/9829	1.30	1.57E-11
GO:0065007	biological regulation	2029/27430	927/9829	1.28	8.67E-20

This table describes second GO terms significantly enriched in core regions based on GeneMerge1.2 software. 36 terms are detected to be significant here. Pop-frec describes the frequency of genes in the population with this term, and CR-frec describes the frequency of genes in the core regions with this term. Ratio is calculated by the comparison of a term within the core regions to that in genome wide. P value here is a Bonferroni corrected P value.

### Pathway Enrichment Analyses

We detected over-represented pathways relevant to genes within detected core regions. Accordingly, 9 out of 213 (4.23%) pathways were found to be significant in the core regions (Table 5), *e.g.*, 471 genes were contributed in the significant ‘metabolic pathways’ (bta01100) which could plausibly be involved in the processes of milk production; The term ‘chemokine signaling pathway’ (bta04062) showed significant over-representation involved in the processes of inflammatory immune response in mammary gland containing 89 genes in all. Other over-represented pathways also reflected some important biological processes, such as: ‘focal adhesion’ (bta04510) participated in cell motility and cell proliferation, ‘spliceosome’ (bta03040) assembled on the mRNA precursor with the function of folding the precursor into a conformation to allow transesterification to proceed.

**Table 5:** Enriched pathway terms when comparing candidate genes to the whole genome using GeneMerge1.2.

Pathway term	Description	Pop-frec	CR-frec	ratio	P value
bta04510	Focal adhesion	192/27430	93/9829	1.35	0.0464
bta03040	Spliceosome	126/27430	67/9829	1.48	0.0107
bta04270	Vascular smooth muscle contraction	122/27430	66/9829	1.51	5.94E-3
bta05322	Systemic lupus erythematosus	189/27430	92/9829	1.36	0.0402
bta04080	Neuroactive ligand-receptor interaction	318/27430	155/9829	1.36	3.00E-4
bta04010	MAPK signaling pathway	266/27430	131/9829	1.37	9.80E-4
bta01100	Metabolic pathways	1081/27430	471/9829	1.22	1.00E-5

This table describes significant pathway terms over-represented in core regions based on GeneMerge1.2 software. Pop-frec describes the frequency of genes in the population with this pathway, and CR-frec describes the frequency of genes in the core regions with this pathway. Ratio is calculated by the comparison of a term within the core regions to that in genome wide. P value here is a Bonferroni corrected P value.

## Discussion

Due to serving as models for basic studies of some important pathways, cattle play a special role in biology (Bovine Genome Sequencing Initiative http://www.genome.gov/Pages/Research/Sequencing/SeqProposals/BovineSEQ). In the past few years, many studies were performed in the field of cattle genetics. For instance, Kaupe et al. [18] genotyped 1748 DNA samples of 38 different Bos taurus and Bos indicus cattle breeds to examine the DGAT1 polymorphism in cattle breeds; another research used Bos taurus and Bos indicus cattle to detect the utilization of low-quality roughage [19]. So far searching for genomic evidence of positive selection in cattle recently has been widely considered as an attractive strategy for identifying functional polymorphisms and providing important insight into the mechanism of evolution [20]. In this study, we carried out EHH tests to detect population-specific selection signatures by using the high density SNP chips in Chinese Holstein and then performed various systems biology analyses to confirm the biological significance for the identified core regions.

Totally, there were 125 core regions been detected potentially harboring selective signals at the significance level of p = 0.01. As Qanbari *et al.* (2010) said, by comparing with shorter extent of homozygositiy of other haplotypes presented in these regions, it can demonstrate the longer extent of homozygosity of some haplotypes were not simply because of low recombination rates but due to the strong recent selection. In a recent study of detecting selective signatures in German Holstein cattle based on EHH tests, a total of 161 regions were identified to exhibit the signals of positive selection. Annotation for these regions also revealed a list of functional candidate genes like *SPATA17, DGAT1, FABP3* and *ABCE1* [9]. Another study carried out by Flori et al. (2009) in three major French dairy cattle breeds, showed 13 highly significant regions under the recent selection. Some of them contained genes that with strong effects on milk production traits, such as *GHR, LAP3* and *ABCG2*. These two researches revealed that most of genes targeted by artificial selection in dairy cattle mainly tended to improve milk production [21]. So, for further probing into the relationships between the identified selection signatures and important traits under selection in this study, we compared these 125 core regions with findings in our previous GWAS studies as well as a candidate gene database for economic traits in dairy cattle. A suite of genes in the identified core regions have been suggested relevant to milk production traits and mastitis. For instance, two core regions overlapped with the region harboring *polycystic kidney disease 2* (*PKD2*) gene, which functioned as a calcium permeable cation channel and had been identified significantly associated with milk protein percentage [12]; *G protein-coupled receptor 20* (*GPR20*) gene located in an overlapped area of 3 extended core regions on chromosome 14, and it is related to milk fat percentage traits. On BTA 6, a region with a haplotype showed the lowest P value (P = 0.0022) hiding the *HECT domain and RLD 3* (*HERC3*) and *polycystic kidney disease 2* (*PKD2*) gene, *HERC3* gene involved in the pathway of ubiquitination mediated proteolysis and it mainly functions as a signal for 26S proteasome dependent protein degradation. Another signal on chromosome 6, which harboring *ATP-binding cassette sub-family G member 2* (*ABCG2*) has been proved as a target of selection [22], [23]. These results indicated that these core regions presented biologic signals associated with milk production performance.

It was notable that the core region harboring *DGAT1* gene merely showed a marginal significance (*p* = 0.057), although *DGAT1* gene has been suggested as being under selection and been reported for many times. This could be due to the higher initial frequency of some beneficial alleles leading to less polymorphism in haplotypes [24]. Another possible reason could be the limitation of SNP density.

In our study, we conducted enrichment analyses for the second GO terms and pathways, which makes use of specifically meaningful annotations rather than uninformative ones and provides insight into regional genomic function [25]. This has been proved to successfully identify over-represented terms for general annotation analyses such as post-genomic analysis, DNA chips and gene networks analysis, and QTL global meta-analysis [26]. In the analyses, we firstly carried out enrichment GO analyses on 418 candidate genes in the database [16]. There were 57 (32.76%) over-represented terms have been found, which also provided a strong indication that genes may be clustered in a manner that reflects their functional association with particular traits. Subsequently, enrichment analyses on genes in the identified core regions were performed and 36 second level GO terms (64.3%) were found to be significantly over-represented in core regions including a total of 2740 functional genes, of which 34 significant terms were coincide with the results of the analyses for 418 candidate genes. These results were consistent with our previous hypothesis that some functional genes associated with milk production traits, as reflected by GO terms could be clustered in core regions compared against the entire genome regions.

We finally analyzed the enriched pathways with the purpose to confirm the biological associations between core regions and quantitative traits. The results of the enriched pathway analyses showed a total of 9 out of 213 (4.23%) pathways were found to be significant in core regions, of which 6 (66.67%) significant pathways were included in the results of candidate genes, confirming that genes involved in functional biological processes. As expected, milk production processes were enriched in core regions, which revealed proofs of multiple genes in a same pathway presenting selective signatures. These results were consistent with the results reported by Qanbari et al. (2010) that the identified candidate genes reflected a panel of pathways such as steroid metabolism and transportation. All these demonstrated the feasibility of EHH test as well as the biological significance of the identified cores based on this method. Findings in our study could help detect functional candidate genes under positive selection for further genetic and breeding studies in Chinese Holstein.

In addition, it was not ignorable that the length of these significant core regions identified by EHH tests was generally large and contained many genes, thus reducing the accuracy of this method; On the other hand, the method of EHH test may lack sensitive for identifying lower-frequency selected alleles [10], resulting in some low-frequency functional genes not included in our results. As for the enriched GO term analyses to detect the distribution of functional genes, we found that the proportion of significant terms were limited. This could be because many of the bovine gene models remain un-annotated. Hence it only roughly reflects our hypothesis that core regions displaying signatures are suggested targets of recent selection. New powerful methods are needed to enhance the power for localizing the source of selection. Due to each single test for selective signatures can provide partial information, we could possibly combine them in a composite likelihood statistic. This idea has already been proved effectively in Human population [10]. Besides, as the differences of allele frequency between populations may be more palpable in the regions harboring causal variants, we can combine with the tests for selective signatures between populations to help us narrow core regions for further accurately positioning the causal variants.

In all, this study detected 125 core regions over the whole genome in Chinese Holstein using the EHH test associated with some bioinformatics analyses. Most of the genes included in these core regions were consistent with reported candidate genes for milk production and mastitis, of which some functional genes classified by GO terms or involved in important pathways of milk production traits were enriched in these identified regions. Our results provided evidence for the exploitability of the core regions identified by EHH tests, and eventually lay a substrate for further studies for targeting causal mutations underlying some important economic traits in Chinese Holstein.
